# 3,3′-Di-*n*-propyl-1,1′-[*p*-phenyl­enebis(methyl­ene)]diimidazolium dibromide

**DOI:** 10.1107/S1600536811026146

**Published:** 2011-07-06

**Authors:** Rosenani A. Haque, S. Fatimah Nasri, Madhukar Hemamalini, Hoong-Kun Fun

**Affiliations:** a School of Chemical Sciences, Universiti Sains Malaysia, 11800 USM, Penang, Malaysia; b X-ray Crystallography Unit, School of Physics, Universiti Sains Malaysia, 11800 USM, Penang, Malaysia

## Abstract

The asymmetric unit of the title compound, C_20_H_28_N_4_^2+^·2Br^−^, consists of half a 3,3′-di-*n*-propyl-1,1′-[*p*-phenyl­enenis(methyl­ene)]diimidazolium cation and a bromide anion. The cation is located on an inversion center and adopts an ⋯*AAA*⋯ *trans* conformation. In the crystal, the cation is linked to the anions *via* weak C—H⋯Br hydrogen bonds.

## Related literature

For details of *N*-heterocyclic carbenes, see: Herrmann *et al.* (1998 ▶); Zhang & Trudell (2000 ▶); Lee *et al.* (2004 ▶). For structures with similar ⋯AAA⋯ *trans* conformations, see: Chen *et al.* (2007 ▶); Cheng *et al.* (2009 ▶).
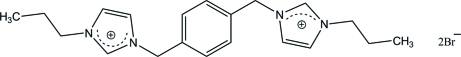


## Experimental

### 

#### Crystal data

C_20_H_28_N_4_^2+^·2Br^−^
*M* *_r_* = 484.28
Monoclinic, 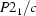

*a* = 8.9420 (2) Å
*b* = 11.2443 (2) Å
*c* = 11.3536 (2) Å
β = 109.716 (1)°
*V* = 1074.64 (4) Å^3^
*Z* = 2
Mo *K*α radiation
μ = 3.78 mm^−1^
*T* = 296 K
0.37 × 0.35 × 0.30 mm

#### Data collection

Bruker SMART APEXII CCD area-detector diffractometer
Absorption correction: multi-scan (*SADABS*; Bruker, 2009 ▶) *T*_min_ = 0.338, *T*_max_ = 0.397
11799 measured reflections
3127 independent reflections
2409 reflections with *I* > 2σ(*I*)
*R*_int_ = 0.030

#### Refinement

*R*[*F* ^2^ > 2σ(*F* ^2^)] = 0.030
*wR*(*F* ^2^) = 0.078
*S* = 1.04
3127 reflections
118 parameters
H-atom parameters constrained
Δρ_max_ = 0.30 e Å^−3^
Δρ_min_ = −0.59 e Å^−3^

### 

Data collection: *APEX2* (Bruker, 2009 ▶); cell refinement: *SAINT* (Bruker, 2009 ▶); data reduction: *SAINT*; program(s) used to solve structure: *SHELXTL* (Sheldrick, 2008 ▶); program(s) used to refine structure: *SHELXTL*; molecular graphics: *SHELXTL*; software used to prepare material for publication: *SHELXTL* and *PLATON* (Spek, 2009 ▶).

## Supplementary Material

Crystal structure: contains datablock(s) global, I. DOI: 10.1107/S1600536811026146/lh5273sup1.cif

Structure factors: contains datablock(s) I. DOI: 10.1107/S1600536811026146/lh5273Isup2.hkl

Supplementary material file. DOI: 10.1107/S1600536811026146/lh5273Isup3.cml

Additional supplementary materials:  crystallographic information; 3D view; checkCIF report

## Figures and Tables

**Table 1:** Hydrogen-bond geometry (Å, °)

*D*—H⋯*A*	*D*—H	H⋯*A*	*D*⋯*A*	*D*—H⋯*A*
C7—H7*A*⋯Br1	0.93	2.77	3.6222 (18)	152

**Fig. 1.**
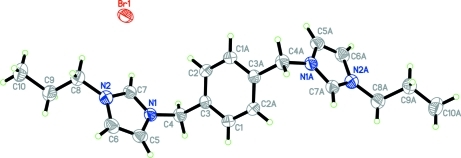
The molecular structure of the title compound, showing 50% probability displacement ellipsoids. Only the unique anion is shown (symmetry code (A): -x, -y+1, -z+1).

**Fig. 2.**
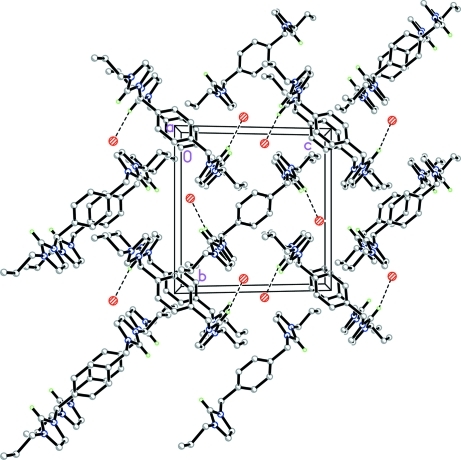
The crystal packing of the title compound, showing weak hydrogen bonds as dashed lines.

## supplementary crystallographic information

### Comment

*N*-Heterocyclic carbene (NHC) ligands have been shown to have wide applicability in coordination chemistry and catalysis. Current research efforts are devoted to the discovery of efficient metal NHC catalysts. For example, chelating palladium complexes of bis(NHC) carbenes have been found to be efficient catalysts in C–C coupling reactions (Herrmann *et al.*, 1998; Zhang & Trudell, 2000). NHC ligands are generally accessible via the deprotonation of imidazolium salts. The preparation of chelating bis(NHC) ligands are also receiving much attention, since they can provide extra air and moisture stability for the metal centers.  Several bis(imidazolium) halides, as bis(NHC) ligand precursors, have been synthesized and structurally characterized by us (Lee *et al.*, 2004). We report here the structure of 3,3'-Di-n-propyl-1,1'-(*p*- phenylenedimethylene)diimidazolium dibromide, (I).

The asymmetric unit of the title compound, consists of a half of the 3,3'-Di-n-propyl-1,1'-(*p*-phenylenedimethylene) diimidazolium cation (located on a crystallographic inversion center) and a bromide anion (Fig. 1).  The cation adopts the ···AAA··· trans conformation in the solid state.  This conformation is the same as that found for the neutral N1,N2-di(2-pyridyl)adipoamide ligand which cocrystallizes with water and 2-{5-[N-(2-Pyridyl)carbamoyl]pentanamido} pyridinium hexafluorophosphate (Chen *et al.*, 2007; Cheng *et al.*, 2009).

In the crystal structure (Fig.2), the cations and anions are linked *via* C7—H7A···Br1 (Table 1) hydrogen bonds.

### Experimental

To a solution of 1,4-bis((1*H*-imidazol-1-yl)methyl)benzene (1.0 g, 4.2 mmol) in 15 ml of acetonitrile, 1-bromopropane (1.0 g, 8.4 mmol) was added. The mixture was refluxed at 363 K for 24 h. The resulting white precipitate was filtered, washed with fresh acetonitrile (2 X 3 ml) and recrystallised from methanol to give colorless crystals. Yield :1.3 g, (93%); m.p: 521–523 K. Crystals suitable for X-ray diffraction studies were obtained by slow evaporation of the salt solution in methanol at ambient temperature.

### Refinement

All hydrogen atoms were positioned geometrically [C–H = 0.93–0.97 Å] and were refined using a riding model, with *U*_iso_(H) = 1.2 or 1.5 *U*_eq_(C). A rotating group model was applied to the methyl groups.

### Crystal data

**Table:**

C_20_H_28_N_4_^2+^·2Br^−^	*F*(000) = 492
*M**_r_* = 484.28	*D*_x_ = 1.497 Mg m^−^^3^
Monoclinic, *P*2_1_/*c*	Mo *K*α radiation, λ = 0.71073 Å
Hall symbol: -P 2ybc	Cell parameters from 4287 reflections
*a* = 8.9420 (2) Å	θ = 2.4–29.8°
*b* = 11.2443 (2) Å	µ = 3.78 mm^−^^1^
*c* = 11.3536 (2) Å	*T* = 296 K
β = 109.716 (1)°	Block, colourless
*V* = 1074.64 (4) Å^3^	0.37 × 0.35 × 0.30 mm
*Z* = 2	

### Data collection

**Table:**

Bruker SMART APEXII CCD area-detector diffractometer	3127 independent reflections
Radiation source: fine-focus sealed tube	2409 reflections with *I* > 2σ(*I*)
graphite	*R*_int_ = 0.030
φ and ω scans	θ_max_ = 30.1°, θ_min_ = 2.4°
Absorption correction: multi-scan (*SADABS*; Bruker, 2009)	*h* = −12→12
*T*_min_ = 0.338, *T*_max_ = 0.397	*k* = −15→15
11799 measured reflections	*l* = −11→16

### Refinement

**Table:**

Refinement on *F*^2^	Primary atom site location: structure-invariant direct methods
Least-squares matrix: full	Secondary atom site location: difference Fourier map
*R*[*F*^2^ > 2σ(*F*^2^)] = 0.030	Hydrogen site location: inferred from neighbouring sites
*wR*(*F*^2^) = 0.078	H-atom parameters constrained
*S* = 1.04	*w* = 1/[σ^2^(*F*_o_^2^) + (0.0366*P*)^2^ + 0.234*P*] where *P* = (*F*_o_^2^ + 2*F*_c_^2^)/3
3127 reflections	(Δ/σ)_max_ < 0.001
118 parameters	Δρ_max_ = 0.30 e Å^−^^3^
0 restraints	Δρ_min_ = −0.59 e Å^−^^3^

### Special details

**Table:**

Geometry. All s.u.'s (except the s.u. in the dihedral angle between two l.s. planes) are estimated using the full covariance matrix. The cell s.u.'s are taken into account individually in the estimation of s.u.'s in distances, angles and torsion angles; correlations between s.u.'s in cell parameters are only used when they are defined by crystal symmetry. An approximate (isotropic) treatment of cell s.u.'s is used for estimating s.u.'s involving l.s. planes.
Refinement. Refinement of F^2^ against ALL reflections. The weighted R-factor wR and goodness of fit S are based on F^2^, conventional R-factors R are based on F, with F set to zero for negative F^2^. The threshold expression of F^2^ > 2σ(F^2^) is used only for calculating R-factors(gt) etc. and is not relevant to the choice of reflections for refinement. R-factors based on F^2^ are statistically about twice as large as those based on F, and R- factors based on ALL data will be even larger.

### Fractional atomic coordinates and isotropic or equivalent isotropic displacement parameters (Å^2^)

**Table:**

	*x*	*y*	*z*	*U*_iso_*/*U*_eq_	
Br1	0.24338 (3)	0.428391 (17)	0.069769 (19)	0.04870 (9)	
N1	0.06730 (17)	0.72483 (12)	0.28704 (13)	0.0340 (3)	
N2	0.28760 (18)	0.76133 (14)	0.25525 (15)	0.0389 (3)	
C1	−0.0905 (2)	0.60075 (16)	0.49714 (17)	0.0358 (4)	
H1A	−0.1508	0.6684	0.4960	0.043*	
C2	0.0537 (2)	0.47667 (16)	0.40181 (16)	0.0350 (4)	
H2A	0.0907	0.4608	0.3360	0.042*	
C3	−0.03759 (19)	0.57747 (14)	0.39781 (16)	0.0316 (3)	
C4	−0.0764 (2)	0.66197 (17)	0.28836 (18)	0.0383 (4)	
H4A	−0.1216	0.6179	0.2110	0.046*	
H4B	−0.1547	0.7193	0.2941	0.046*	
C5	0.1346 (2)	0.82204 (16)	0.35896 (17)	0.0401 (4)	
H5A	0.0933	0.8639	0.4116	0.048*	
C6	0.2712 (2)	0.84497 (16)	0.33885 (18)	0.0427 (4)	
H6A	0.3420	0.9061	0.3747	0.051*	
C7	0.1620 (2)	0.68965 (16)	0.22476 (17)	0.0370 (4)	
H7A	0.1434	0.6259	0.1695	0.044*	
C8	0.4185 (2)	0.75295 (18)	0.2048 (2)	0.0456 (4)	
H8A	0.4123	0.6772	0.1625	0.055*	
H8B	0.5189	0.7557	0.2732	0.055*	
C9	0.4132 (2)	0.85232 (19)	0.1143 (2)	0.0469 (5)	
H9A	0.4181	0.9283	0.1558	0.056*	
H9B	0.3139	0.8488	0.0446	0.056*	
C10	0.5517 (3)	0.8419 (2)	0.0657 (2)	0.0586 (6)	
H10A	0.5471	0.9058	0.0085	0.088*	
H10B	0.5457	0.7672	0.0234	0.088*	
H10C	0.6499	0.8462	0.1347	0.088*	

### Atomic displacement parameters (Å^2^)

**Table:**

	*U*^11^	*U*^22^	*U*^33^	*U*^12^	*U*^13^	*U*^23^
Br1	0.06110 (15)	0.03539 (11)	0.05110 (14)	0.00544 (9)	0.02086 (10)	0.00409 (8)
N1	0.0411 (8)	0.0308 (7)	0.0319 (7)	−0.0010 (6)	0.0149 (6)	0.0013 (6)
N2	0.0424 (8)	0.0354 (7)	0.0417 (9)	−0.0021 (6)	0.0176 (7)	−0.0004 (6)
C1	0.0370 (9)	0.0318 (8)	0.0416 (10)	0.0037 (7)	0.0173 (8)	0.0001 (7)
C2	0.0386 (9)	0.0355 (8)	0.0352 (9)	0.0020 (7)	0.0181 (8)	−0.0010 (7)
C3	0.0301 (8)	0.0311 (8)	0.0337 (8)	−0.0039 (6)	0.0109 (7)	0.0001 (7)
C4	0.0357 (9)	0.0406 (9)	0.0382 (10)	−0.0003 (7)	0.0119 (7)	0.0036 (8)
C5	0.0552 (11)	0.0329 (8)	0.0333 (9)	0.0016 (8)	0.0165 (8)	−0.0023 (7)
C6	0.0522 (11)	0.0349 (9)	0.0397 (10)	−0.0072 (8)	0.0138 (9)	−0.0035 (8)
C7	0.0456 (10)	0.0319 (8)	0.0361 (9)	−0.0015 (7)	0.0171 (8)	−0.0009 (7)
C8	0.0416 (10)	0.0456 (10)	0.0545 (12)	0.0018 (8)	0.0224 (9)	0.0055 (9)
C9	0.0471 (11)	0.0496 (11)	0.0484 (11)	0.0004 (9)	0.0219 (9)	0.0047 (9)
C10	0.0566 (13)	0.0693 (15)	0.0598 (14)	−0.0037 (11)	0.0326 (11)	0.0048 (12)

### Geometric parameters (Å, °)

**Table:**

N1—C7	1.333 (2)	C4—H4B	0.9700
N1—C5	1.376 (2)	C5—C6	1.341 (3)
N1—C4	1.471 (2)	C5—H5A	0.9300
N2—C7	1.330 (2)	C6—H6A	0.9300
N2—C6	1.379 (2)	C7—H7A	0.9300
N2—C8	1.470 (2)	C8—C9	1.508 (3)
C1—C3	1.387 (2)	C8—H8A	0.9700
C1—C2^i^	1.388 (3)	C8—H8B	0.9700
C1—H1A	0.9300	C9—C10	1.521 (3)
C2—C1^i^	1.388 (3)	C9—H9A	0.9700
C2—C3	1.389 (2)	C9—H9B	0.9700
C2—H2A	0.9300	C10—H10A	0.9600
C3—C4	1.509 (2)	C10—H10B	0.9600
C4—H4A	0.9700	C10—H10C	0.9600
			
C7—N1—C5	108.76 (15)	C5—C6—N2	107.47 (16)
C7—N1—C4	125.17 (15)	C5—C6—H6A	126.3
C5—N1—C4	125.78 (14)	N2—C6—H6A	126.3
C7—N2—C6	108.44 (15)	N2—C7—N1	108.29 (15)
C7—N2—C8	124.99 (16)	N2—C7—H7A	125.9
C6—N2—C8	126.56 (16)	N1—C7—H7A	125.9
C3—C1—C2^i^	120.18 (16)	N2—C8—C9	111.85 (16)
C3—C1—H1A	119.9	N2—C8—H8A	109.2
C2^i^—C1—H1A	119.9	C9—C8—H8A	109.2
C1^i^—C2—C3	120.77 (16)	N2—C8—H8B	109.2
C1^i^—C2—H2A	119.6	C9—C8—H8B	109.2
C3—C2—H2A	119.6	H8A—C8—H8B	107.9
C1—C3—C2	119.05 (16)	C8—C9—C10	110.34 (17)
C1—C3—C4	120.26 (15)	C8—C9—H9A	109.6
C2—C3—C4	120.68 (15)	C10—C9—H9A	109.6
N1—C4—C3	110.61 (14)	C8—C9—H9B	109.6
N1—C4—H4A	109.5	C10—C9—H9B	109.6
C3—C4—H4A	109.5	H9A—C9—H9B	108.1
N1—C4—H4B	109.5	C9—C10—H10A	109.5
C3—C4—H4B	109.5	C9—C10—H10B	109.5
H4A—C4—H4B	108.1	H10A—C10—H10B	109.5
C6—C5—N1	107.04 (16)	C9—C10—H10C	109.5
C6—C5—H5A	126.5	H10A—C10—H10C	109.5
N1—C5—H5A	126.5	H10B—C10—H10C	109.5
			
C2^i^—C1—C3—C2	−0.7 (3)	N1—C5—C6—N2	−0.3 (2)
C2^i^—C1—C3—C4	−179.54 (16)	C7—N2—C6—C5	0.5 (2)
C1^i^—C2—C3—C1	0.7 (3)	C8—N2—C6—C5	179.06 (17)
C1^i^—C2—C3—C4	179.54 (16)	C6—N2—C7—N1	−0.4 (2)
C7—N1—C4—C3	92.7 (2)	C8—N2—C7—N1	−179.02 (16)
C5—N1—C4—C3	−80.3 (2)	C5—N1—C7—N2	0.2 (2)
C1—C3—C4—N1	110.19 (18)	C4—N1—C7—N2	−173.88 (15)
C2—C3—C4—N1	−68.6 (2)	C7—N2—C8—C9	107.5 (2)
C7—N1—C5—C6	0.1 (2)	C6—N2—C8—C9	−70.9 (2)
C4—N1—C5—C6	174.13 (16)	N2—C8—C9—C10	179.05 (18)

Symmetry codes: (i) −*x*, −*y*+1, −*z*+1.

### Hydrogen-bond geometry (Å, °)

**Table:**

*D*—H···*A*	*D*—H	H···*A*	*D*···*A*	*D*—H···*A*
C7—H7A···Br1	0.93	2.77	3.6222 (18)	152
